# Feline mammary basal-like adenocarcinomas: a potential model for human triple-negative breast cancer (TNBC) with basal-like subtype

**DOI:** 10.1186/1471-2407-13-403

**Published:** 2013-09-03

**Authors:** David A Wiese, Tuddow Thaiwong, Vilma Yuzbasiyan-Gurkan, Matti Kiupel

**Affiliations:** 1 Department of Pathology, McLaren Regional Medical Center, Flint, MI, USA; 2 Diagnostic Center for Population and Animal Health, Michigan State University, East Lansing, MI, USA; 3 Comparative Medicine and Integrative Biology, Michigan State University, East Lansing, MI, USA; 4 Department of Pathobiology and Diagnostic Investigation, Michigan State University, East Lansing, MI, USA

**Keywords:** Basal phenotype, BRCA, Feline, Mammary adenocarcinoma, Triple negative

## Abstract

**Background:**

Breast cancer is one of the leading causes of cancer deaths. Triple-negative breast cancer (TNBC), an immunophenotype defined by the absence of immunolabeling for estrogen receptor (ER), progesterone receptor (PR) and HER2 protein, has a highly aggressive behavior. A subpopulation of TNBCs exhibit a basal-like morphology with immunohistochemical positivity for cytokeratins 5/6 (CK5/6) and/or epidermal growth factor receptor (EGFR), and have a high incidence of BRCA (breast cancer susceptibility) mutations. Feline mammary adenocarcinomas (FMAs) are highly malignant and share a similar basal-like subtype. The purpose of this study was to classify FMAs according to the current human classification of breast cancer that includes evaluation of ER, PR and HER2 status and expression of basal CK 5/6 and EGFR. Furthermore, we selected triple negative, basal-like FMAs to screen for BRCA mutations similar to those described in human TNBC.

**Methods:**

Twenty four FMAs were classified according to the current human histologic breast cancer classification including immunohistochemistry (IHC) for ER, PR HER2, CK5/6 and EGFR. Genetic alteration and loss of heterozygosity of BRCA1 and BRCA2 genes were analyzed in triple negative, basal-like FMAs.

**Results:**

IHC for ER, PR and HER2 identified 14 of the 24 (58%) FMAs as a triple negative. Furthermore, 11of these 14 (79%) triple negative FMAs had a basal-like subtype. However, no genetic abnormalities were detected in BRCA1 and BRCA2 by direct sequencing and loss of heterozygosity analysis.

**Conclusion:**

FMAs are highly aggressive neoplasms that are commonly triple negative and exhibit a basal-like morphology. This is similar to human TNBC that are also commonly classified as a basal-like subtype. While sequencing of a select number of triple negative, basal-like FMAs and testing for loss of heterozygosity of BRCA1 and BRCA2 did not identify mutations similar to those described in human TNBC, further in-depth evaluation is required to elucidate a potential role of BRCA in the tumorigenesis of triple negative, basal-like FMAs. The strong similarities in clinical behavior, morphology and IHC phenotype suggest that triple negative, basal-like FMAs may be a suitable spontaneous animal model for studying novel therapeutic approaches against human basal-like TNBC.

## Background

Breast cancer is the most common neoplasm in women, with approximately 230,480 new cases and 39,520 deaths occurring in 2011 in the United States [1]. The disease is complicated by its diverse pathogenesis, and by large phenotypic variations. Classification systems based entirely on morphologic criteria are yielding to new classifications based that also include molecular and immunophenotypic markers [2] to more accurately diagnose and prognostic breast cancer. The most recent classification system classifies human breast cancers into at least five prognostically significant subtypes, including estrogen receptor-positive luminal A and B subtypes, HER2 overexpression subtype, normal breast-like and basal-like subtype [3]. A variety of immunohistochemical markers have been proposed to define a basal-like subtype [4,5]. Expression of one or more high molecular weight basal cytokeratins (CK5/6, CK14, and CK-17) and/or epidermal growth factor receptor (EGFR) are most commonly accepted to identify basal-like differentiation [6]. Furthermore, a large percentage of basal-like breast cancers lack expression of estrogen receptor (ER) and progesterone receptor (PR), as well as HER2 (‘triple negative’ immunophenotype) [7]. While basal-like breast cancer has the worst clinical outcome measured in time to development of distant metastasis [8], there is still some controversy on the histologic classification of this subtype regarding whether triple negative status is required for making the diagnosis of basal-like carcinoma. Some investigations have indicated that 77% of basal-like breast cancers are triple negative (TNBC) and 71%-91% of TNBC have basal-like phenotype [9]. Research has focused especially on TNBC since they tend to have a poor prognosis with a high risk of distant metastasis and death within the first 3–5 years after the diagnosis [10] and no effective treatment is currently available. Furthermore, TNBC have a high incidence in younger, premenopausal patients with higher prevalence in African-American women, and are associated with germ-line BRCA1 mutations [11].

Feline mammary tumors are the third most common tumors in the cat, and most tumors are malignant. Feline mammary tumors are predominantly adenocarcinomas [12], with sarcomas and other non-epithelial tumors occurring less frequently. Feline mammary adenocarcinomas (FMAs) are most commonly classified as in-situ or invasive carcinomas, the latter consisting of tubulopapillary, cribriform and anaplastic types. Less common varieties include spindle cell carcinoma, lipid rich carcinoma, mucinous carcinoma and tumors with squamous differentiation [13]. However, little work has been done to characterize feline mammary tumors based on new concepts of breast cancer classification as applied to human tumors.

Various parameters have been applied for prognostication of FMAs including the size of tumors, histological grading, lymph node involvement, and expression of proliferation markers [14]. Due to the similarities between human breast cancer and FMAs in regard to epidemiology, clinical behavior, pattern of metastasis, and histological features, various studies have been suggested that FMAs are a potential model of human breast cancer [15,16].

Aberrant expression of hormonal receptors ER and PR and overexpression of HER2 have also been described in feline mammary tumors. The majority of feline mammary tumors are ER-negative [17] and lack HER2 overexpression [18]. In addition, the majority of feline mammary carcinomas are ER- and PR-negative as studied by ligand-binding assay [19,20] as well as by immunohistochemistry [21-23]. Controversy exists with respect to the proportion that express HER2 [18,24,25]. However, only a few studies have evaluated feline mammary tumors as a potential model for human TNBC [26].

In humans, breast cancer-susceptibility 1 *(BRCA1)* and breast cancer-susceptibility 2 (*BRCA2)* genes have been found to be mutated in a large number of early-onset breast or ovarian cancers [27,28]. Somatic loss of heterozygosity (LOH), which is the loss of a normal functioning allele at the heterozygous locus, is one of the genetic alterations associated with breast cancer initiation and progression [29]. Especially in patients that carry germ line mutations in tumor suppressor genes such as *BRCA1* or *BRCA2*, acquired LOH of the wild-type allele, corresponding to recessive loss-of-function mutations, will lead to hereditary breast cancer [30]. Studies in humans have shown that breast cancer with *BRCA1* mutations and less commonly *BRCA2* mutations display predominantly a triple negative immunophenotype with distinct basal-like subtype [31,32]. More than 75% of tumors arising in women carrying a *BRCA1* mutation display a triple-negative immunophenotype, a basal-like subtype, or both [33].

In the current study, a series of twenty four feline mammary adenocarcinomas were evaluated according to the current human histologic breast cancer classification, and the TNBC immunophenotype and basal-like subtypes were identified by immunohistochemical expression of ER, PR, and HER2, as well as basal cytokeratins CK5/6 and EGFR. In addition the histologic growth pattern, tumor grade, presence of necrosis, presence of microcalcification, and lymphocytic reaction associated with the tumor were evaluated. Further investigation of genetic alteration and loss of heterozygosity in *BRCA1* and *BRCA2* were conducted in feline triple negative, basal-like mammary adenocarcinomas to determine whether the presence of *BRCA1/BRCA2* mutations was associated with this phenotype similar to the observations in human breast cancer.

## Methods

### Cases

Twenty four cases of feline mammary adenocarcinomas were retrieved from the archives of the Diagnostic Center for Population and Animal Health at Michigan State University. All cases had been submitted over a 3 year time span from 01/01/2004 until 12/12/2007. Biopsy samples from 24 cats were included in the study based on a previously confirmed histopathological diagnosis of FMAs, sufficient archival material in high quality (absence of extensive necrosis or autolysis) and histologically unremarkable mammary tissue in the same or in additional paraffin blocks. Mean age at the time of diagnosis was 12 years (range, 6 to 19 years). Of the 24 cats, 18 were spayed female, 4 were intact female, 1 was castrated male, and 1 was of unidentified gender. These cases represented approximately 80% of malignant feline mammary tumors over the archival time span.

### Histological grading

Representative formalin-fixed paraffin-embedded tissue blocks were recut at a thickness of 4 microns and were stained routinely with hematoxylin and eosin for histologic evaluation. Tumors were histopathologically classified according to the World Health Organization (WHO) [13], and the three most predominant growth patterns were recorded. Tumor grading was performed by the Nottingham modification of the Scarff-Bloom-Richardson grading system as reported by Elston and Ellis [34]. Briefly, this method assigns a score of 1, 2 or 3 for each of three histologic or cytologic features, including nuclear grade, extent of tubule formation and mitotic rate per 10 high power fields (0.44 mm^2^ field diameter). The scores for each of these parameters are then summed to assign the tumor grade, with scores of 3, 4 or 5 considered grade I, 6 and 7 grade II, and 8 and 9 grade III.

The presence or absence of tumor necrosis was noted, and the presence or absence of calcium hydroxyapatite microcalcifications, detected as distinct purple densities visible with light microscopy on H&E staining, was recorded. Specimens were also polarized to evaluate for possible calcium oxalate microcalcifications, an optically clear but refractile form of microcalcification not usually demonstrable by routine light microscopy.

Tumors were also evaluated for the presence of associated lymphocytic infiltration, recorded by location as peritumoral or intratumoral. Lymphocytic infiltrates were scored as 0 (negligible or absent infiltration), 1+ (few lymphoid aggregates associated with tumor), 2+ (moderate lymphoid aggregates or infiltrates typically seen in 20 to 50% of the tumor), or 3+ (brisk lymphoid aggregates or infiltrates associated with more than 50% of the tumor).

### Immunohistochemistry

Serial sections of each tumor were immunohistochemically labeled for ER using the CONFIRM anti-ER (SP1) rabbit monoclonal antibody, and for PR using the CONFIRM anti-PR (1E2) rabbit monoclonal antibody (both Ventana Medical Systems, Inc, Tucson, AZ). The antibodies were applied for 32 minutes at 37°C for ER, and at 16 minutes at 37°C for PR according to vendor protocol, using the Ventana BenchMark XT automated immunostainer with the Ventana iView detection Kit (Ventana Medical Systems, Inc, Tucson, AZ). ER and PR immunohistochemical labeling was evaluated by recording labeling intensity, location and estimated percentage of positive cells, based on evaluation of a minimum of twenty high power (400×) fields. Labeling intensity was recorded as 0 (no labeling), 1+ (weak labeling above background), 2+ (moderate labeling), or 3+ (strong labeling) as previously described [17].

Additional sections were also labeled for HER2 using the PATHWAY anti-HER2/neu rabbit monoclonal antibody directed against the internal domain of the c-erbB-2 oncoprotein (Ventana Medical Systems, Inc, Tucson, AZ). The antibody was applied for 32 minutes at 37°C according to vendor protocol using the Ventana BenchMark XT automated immunostainer with iView Detection Kit (Ventana Medical Systems, Inc, Tucson, AZ). Immunohistochemical labeling for HER2 was evaluated according to the HercepTest™, recording labeling intensity and location, and estimated percentage of positive cells, also based on a minimum of twenty high power (400×) fields. Specifically, labeling intensity was recorded as 0 (no labeling), 1+ (weak, incomplete membranous labeling), 2+ (moderate, complete membranous labeling), or 3+ (strong, complete membranous labeling) as previously described [17]. Scores 2 and 3 were considered to indicate overexpression, scores 0 and 1 were considered as negative.

To evaluate tumors for features of basal-like subtype, additional sections were immunolabeled for CK5/6 (clone EP1601Y; Cell Marque, Rocklin, Ca), and for epidermal growth factor receptor (Ventana CONFIRM anti-EGFR (5B7) rabbit monoclonal primary antibody (Ventana Medical Systems, Inc, Tucson, AZ), both applied for 16 minutes at 37°C also according to vendor protocol using the Ventana BenchMark XT automated immunostainer with iView Detection Kit (Ventana Medical Systems, Inc, Tucson, AZ). For CK5/6 and EGFR, sections were also evaluated for labeling intensity and location and estimated percentage of positive cells, with labeling intensity recorded as 0 (no labeling), 1+ (weak labeling), 2+ (moderate labeling), or 3+ (strong, intense labeling) [4].

Feline uterus sections were used as positive controls for ER and PR, and a human breast carcinoma was used as positive control for over-expression of HER2. Adjacent normal mammary tissue was used as internal positive controls for CK5/6 and EGFR. For negative controls the primary antibodies were replaced by homologous non-immune sera. All positive and negative controls gave the expected features in all of the evaluation.

### DNA isolation and amplification of BRCA1 and BRCA2

Genomic DNA extracted from paraffin-embedded tissue sections obtained from 11 samples of triple negative, basal-like subtype adenocarcinomas was amplified of normal and tumor tissue following a published protocol [35]. To select population of neoplastic cells from basal-like tumors, immunohistochemistry for CK5/6 and EGFR were used to locate the basal-like subtype area, and only immunopositive non-necrotic areas were collected for DNA extraction. Primers were designed to detect BRCA1 and BRCA2 mutations according to mutations reported by Musolino et al., 2007 [36]. PCR product was visualized on agarose electrophoresis and submitted for both directions sequencing at the Research Technology Support Facility (RTSF), MSU. Sequences were determined on an ABI PRISM® 3730 Genetic Analyzer (Applied Biosystem, CA). Twenty five genomic DNA samples extracted from normal skin collected at routine necropsy from 25 cats that had diseases unrelated to cancer were used as control.

### Evaluation of loss of heterozygosity in BRCA 1and BRCA2

Loss of heterozygosity (LOH) at the BRCA1 and BRCA2 loci was evaluated by using M13-labeling PCR amplification of microsatellite markers at the 5′ and 3′ of both genes. Microsatellites from normal and tumor DNA were amplified using PCR with M13 tailed primers. The forward primer for each primer pair was synthesized with an additional modified 20-bp M13 tailed added to the 5′end. An M13 primer that has the same sequence and that directly labelled to the fluorescence, 6-FAM, was used as a label primer for the detection [37]. Standard PCR reactions were prepared in a 25-μl total volume using 1 μl extracted DNA, 5 pmol of labeled M13 primer, 0.5 pmol of M13 tailed forward primer, 5 pmol unlabeled reverse primer, and 0.625 U of GoTaq® DNA polymerase (Promega, Madison, WI). Cycling conditions were as follows: 40 cycles at 95°C for 1 minute, 59°C for 2 minute, and 72°C for 3 minute; and 72°C for 5 minutes. Amplified products were analyzed by agarose gel electrophoresis on a 2% agarose gel. Fluorescence visualization was performed using Typhoon 9200 Variable Mode Image (GE Healthcare, Pittsburgh, PA). PCR products were further submitted for sequencing using capillary electrophoresis on an ABI PRISM® 3130 Genetic Analyzer (Applied Biosystems, CA). Allelic imbalance was determined using the normalized allelic ratio equation [LOH ratio = (D1)(N2)/(D2)(N1)], where D1 and D2 are the heights of the smaller and larger allelic peaks, respectively, fromthe duct and N1 and N2 are the heights of the allelic peaks from surrounded normal tissue DNA [38]. Allelic imbalance was noted if the area under the target size peak in the basal-like tumor DNA was altered (increase or decrease) by 50% or more, after compared to peak area in the surrounding non-tumor DNA. Samples showing homozygosity result on agarose gel electrophoresis were classified as non-informative.

## Results

### Histological grading

On histopathologic review (Table 1), 3 of the 24 FMAs were fairly pure papillary carcinomas with well-defined papillary structures identified through a majority of the tumors. The remaining 21 FMAs showed a variety of growth patterns. Most displayed admixed components of tubulopapillary, cribriform and solid growth patterns. In addition, cystic areas were common and were seen in seven of the cases.

**Table 1:** Histologic characteristics of feline mammary adenocarcinomas

**Case**	Patterns*	**Nuclear grade**	**% Tubules**	**Mitoses/ 10 HPF**	**Grade**	**Necrosis**	**Micro-calcifications**	Lymphocytie infiltrates**
1	CY, SO, CR	2	60	45	2	Y	N	PT 1
2	SO, TU	2	80	47	2	Y	N	0
3	TU, SO, PA	2	70	54	2	Y	N	PT 2
4	TU, PA	1	80	7	1	Y	Y	PT 1
5	TU, SO	2	70	65	2	Y	N	PT 1
6	SO, TU, CY	2	50	48	2	Y	N	0
7	SO, PA	3	5	20	3	N	N	PT 2
8	TU, CR	2	90	12	2	Y	N	0
9	SO, CY	3	5	56	3	Y	N	PT 1
10	TU, SO, CY	3	80	22	3	Y	N	PT 1
11	TU	3	90	25	2	Y	N	PT 1
12	TU, SO	3	75	18	2	Y	N	PT 1
13	SO, CY	3	10	28	3	Y	N	0
14	TU, SO	3	60	70	3	N	N	0
15	TU	3	90	25	2	Y	N	PT 1
16	CY, TU	2	70	62	2	Y	Y	0
17	PA	2	90	12	2	Y	N	0
18	TU, SO	2	70	45	2	Y	N	0
19	PA	1	90	12	1	N	N	0
20	PA, TU, CY	2	80	42	2	Y	N	PT 2
21	PA	2	90	24	2	Y	N	PT 2
22	SO, TU	2	20	12	2	Y	N	PT 1
23	TU, PA	3	70	52	3	Y	N	PT 1
24	SO, CY	3	5	90	3	Y	N	PT 2

*CY cystic, *SO* solid, *TU* tubular, *PA* papillary.

**PT peritumoral.

According to the histologic characteristics, the FMAs had a low nuclear grade in 3 cases, an intermediate nuclear grade in 12 cases and a high nuclear grade in 9 cases. There was scant tubule formation in 3 cases, intermediate tubule formation in 10 cases and prominent tubule formation in 11 cases. Only one FMA had an intermediate mitotic rate, with 23 having high mitotic rates (>10 figures). In fact, the mitotic rate was typically very high with a mean mitotic rate of 33 and a median of 27 mitoses per 10 high power fields (0.44 mm^2^ field diameter). Based on the overall tumor grade according to the combined scores for nuclear grade, tubule formation and mitotic rate, 2 FMAs were well differentiated (grade 1) tumors, 15 moderately differentiated (grade II) tumors, and 7 poorly differentiated (grade 3).

Tumor necrosis was common, with 21 of the 24 FMAs exhibiting significant (>10%) areas of necrosis, whereas 3 adenocarcinomas were devoid of significant necrosis. Microcalcifications were identified within only 2 FMAs, with the other 22 FMAs having no microcalcifications. In both of the cases with microcalcification, the deposits were identified by light microscopy, representing calcium hydroxyapatite. Polarized light microscopy showed no additional calcium oxalate microcalcifications in any of the tumors examined. Lymphocytic infiltrates associated with tumor were present in 15 of the 24 (62.5%) FMAs, and all had a 1+ to 2+ peritumoral distribution.

### Immunohistochemistry-based molecular subtype classification

Estrogen receptor labeling was present in 2 of the 24 FMAs (8.3%) (Table 2). Labeling was weak (1+) and was present in 10% and 40% of the tumor nuclei respectively. Progesterone receptor labeling (Table 2) was present in 7 of the 24 tumors (29.2%). The labeling intensity was weak (1+) in 5 cases, moderate (2+) in 1 case, and strong (3+) in 1 case, with positive nuclei ranging from 5% to 90%, the latter in the case with strong nuclear labeling. No cases expressed both estrogen and progesterone receptors. HER2 was overexpressed in 4 of the 24 FMAs (Table 2). Labeling was moderate (2+) in all four cases, two with 20% and 40% of cells with cytoplasmic/membrane labeling, one with membranous labeling in 20% of the cells, and one with membranous labeling in 30% of the papillary components. In our series, 14 (58.3%) tumors were triple negative (ER-, PR-, and HER2-).

**Table 2:** Immunohistochemical staining results of ER/PR/HER2, CK5/6, and EGFR

		**ER**			**PR**			**HER2**			**CK5**			**EGFR**	
**Case**	**Intensity**	**Location**	**Percent**	**Intensity**	**Location**	**Percent**	**Intensity**	**Location**	**Percent**	**Intensity**	**Location**	**Percent**	**Intensity**	**Location**	**Percent**
1			0	1	N*	5			0	3	M, C*	100			0
***2***			***0***			***0***			***0***	***3***	***M, C***	***100***			***0***
3			0			0	2	C, M	20	3	M	20	2	C, M	80
4	1	N	40			0	2	M	20	3	M	100	2	M	15
5	1	N	10			0			0	3	M	30	2	M	50
***6***			***0***			***0***			***0***	***3***	***C, M***	***40***	***1***	***M***	***5***
***7***			***0***			***0***			***0***	***3***	***M, C***	***80***	***1***	***M***	***60***
8			0			0			0			0			0
***9***			***0***			***0***			***0***	***3***	***C, M***	***25***	***1***	***C, M***	***30***
10			0	1	N	5			0	3	C, M	5	1	C	10
11			0	3	N	90			0			0			0
12			0	2	N	80			0			0	1	M	5
13			0	1	N	10	2	C, M	40	3	M	80	1	M, C	15
***14***			***0***			***0***			***0***	***3***	***M***	***100***			***0***
***15***			***0***			***0***			***0***	***3***	***M, C***	***70***	***1***	***C, M***	***30***
16			0	1	N	30			0	3	M	50	2	C, M	50
***17***			***0***			***0***			***0***			***0***	***2***	***M, C***	***30***
18			0			0			0			0			0
***19***			***0***			***0***			***0***	***3***	***M, C***	***90***	***2***	***M***	***90***
***20***			***0***			***0***			***0***	***3***	***M***	***90***	***1***	***M***	***80***
21			0	1	N	5	2	M	30			0			0
***22***			***0***			***0***			***0***	***3***	***M***	***20***	***1***	***C, M***	***70***
23			0			0			0			0			0
***24***			***0***			***0***			***0***	***3***	***M***	***70***			***0***

*N nuclear, *C* cytoplasmic, *M* membranous.

Feline triple-negative, basal-like adenocarcinomas (case numbers 2, 6, 7, 9, 14, 15, 17, 19, 20, 22, 24 in italicized boldface) were submitted for genetic analysis.

**Figure 1:**
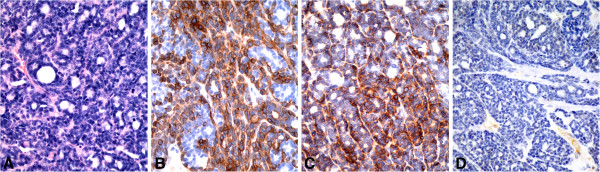
**Identification of triple negative basal-like subtype (ER-, HER2-, CK5/6+ and/or EGFR+).** Sections of feline mammary adenocarcinoma with basal-like **(A)** subtype were CK5/6 **(B)**and EGFR positive **(C)**, but negative for estrogen receptor **(D)**, progesterone receptor and HER2).

Immunolabeling for CK5/6 (Table 2) was identified in the myoepithelial cells lining normal and hyperplastic mammary ducts as well as in hair follicles and portions of epidermal tissues, these structures serving as internal controls. Of the 24 FMAs 17 (71%) had uniformly strong (3+) positive labeling of tumor cells (Figure 1B); 11 of these had 50% or more positive tumor cells, the other 6 cases showing similar strong labeling that varied from 5% to 40% of positive tumor cells. For 17 positive cases, labeling was seen in an average of 63% of the tumor cells. In 2 of the cases, with 20% and 30% positive tumor cells, the pattern appeared clonal, with intense reaction in only a portion of the tumor. In the other cases, the labeling was irregular and diffuse throughout the tumors. Immunolabeling for EGFR (Table 2) similarly showed moderate to strong positive (2 + −3+) labeling in myoepithelial cells of normal and hyperplastic ducts and in incidental sebaceous glands, these again serving as internal controls. Of the 24 FMAs 15 (62.5%) had EGFR positive tumor cells (Figure 1C) with weak (1+) labeling in 9 cases (60%) and moderate (2+) labeling in 6 cases (40%). The labeling was membranous in distribution with 9 cases showing some degree of cytoplasmic labeling as well. Immunolabeling was observed in 50% or more of the tumor cells in 7 cases (47%), with 9 FMAs having 5% to 30% positive tumor cells. For the positive cases labeling was seen in an average of 41% of the tumor cells. Positive labeling appeared diffuse and irregular in the tumors, without the clonal pattern noted in two of the cases when labeled with CK5/6. Thirteen of the 24 FMAs (54%) were positive for both CK5 and EGFR, while 6 (25%) were positive for either CK5/6 or EGFR; the remaining 5 cases (21%) were negative for both CK5/6 and EGFR.

**Figure 2:**
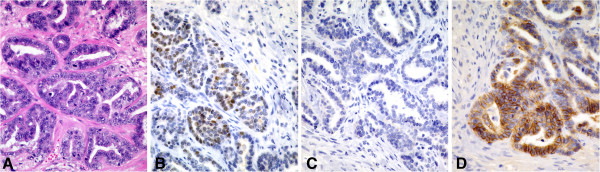
**Identification of estrogen receptor-positive luminal A subtype (ER+, PR + or PR-, HER2-).** Sections of feline mammary adenocarcinoma with tubular **(A)** and solid morphology were estrogen receptor positive **(B)** and HER2 negative **(C)**, but focally positive for EGFR **(D)**.

**Figure 3:**
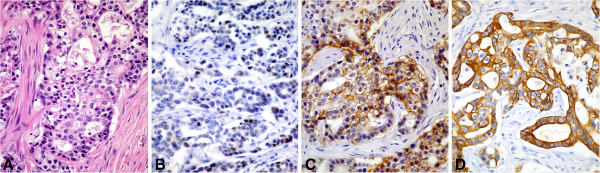
**Identification of estrogen receptor-positive luminal B subtype (ER+, PR + or PR-, HER2+).** Sections of feline mammary adenocarcinoma with tubular **(A)** and papillary morphology were estrogen receptor positive **(B)** and HER2 positive **(C)**, but positive for CK5/6 **(D)**.

**Figure 4:**
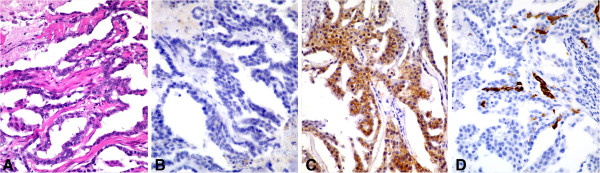
**Identification of HER2 overexpression subtype (ER-, HER2+).** Sections of feline mammary adenocarcinoma with tubular and papillary **(A)** and solid morphology were estrogen receptor negative **(B)** and HER2 positive **(C)**, but focally positive for CK5/6 **(D)**.

Based on expression of ER, PR, HER2, CK5/6, and EGFR, tumor subtypes were immunohistochemically classified as estrogen receptor-positive luminal A (ER+, PR + or PR-, HER2-), estrogen receptor-positive luminal B (ER+, PR + or PR-, HER2+), HER2 overexpression (ER-, PR + or PR-, HER2+), and basal-like (CK5/6+ and/or EGFR+). In our series, the distribution of tumor molecular subtypes was: 1 (4.2%) estrogen receptor-positive luminal A (case 5 in Table 2, Figure 2A-D), 1 (4.2%) estrogen receptor-positive luminal B (case 4 in Table 2, Figure 3A-D), 1 (4.2%) HER2 overexpression (case 3 in Table 2, Figure 4A-D), and 19 (79.2%) basal-like (Table 2, Figure 1A-D).

### Detection of TNBC with basal-like subtype

Eleven of the 14 FMAs (79%) that were negative for estrogen receptor, progesterone receptor and HER2 (triple negative) exhibited a basal-like subtype, 7 of these 14 FMAs were positive for both CK5/6 and EGFR, and four were positive for either CK5/6 or EGFR. Eight of the 24 FMAs (33%) were positive for either CK5/6 or EGFR or both, but had positive labeling for at least one of the following: HER2 (1 case), estrogen receptor (1 case), progesterone receptor (4 cases) or multiple receptors (2 cases), thereby precluding assignment of these tumors as true basal-like subtype TNBCs.

### Genetic analysis

**Figure 5:**
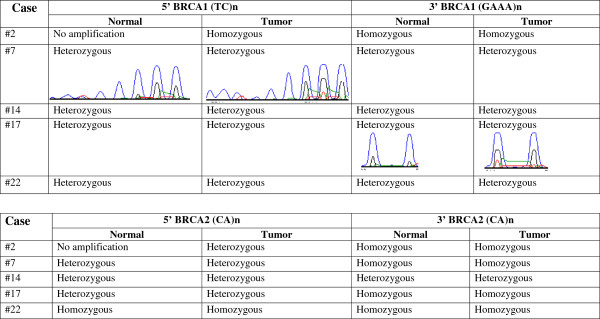
**Detection of loss of heterozygosity in BRCA1 and BRCA2.** No allelic losses were detected in triple negative tumors exhibiting a basal-like phenotype.

Further investigation of genetic alterations in BRCA1 and BRCA2 was performed. Only five of the 11 DNA samples from TNBCs with basal like subtype could be amplified. Examination of BRCA1/BRCA2 sequences revealed missense mutations and polymorphisms, but there were no mutations or polymorphisms specific to the tumors compared to normal feline DNA. No abnormality in *BRCA1* and *BRCA2* alleles was detected (Figure 5. Missense mutations and polymorphisms as compared to the established cat genome (Genome browser: http://genome.ucsc.edu/) were identified in the BRCA1 or BRCA2 gene in triple-negative basal-like FMAs. However, these genetic alterations were not specific for FMAs and were also identified in control DNA obtained from normal cats.

## Discussion

This study was designed to investigate FMAs as a potential natural model for human basal-like TNBC. FMAs are typically aggressive with a significant propensity to metastasize and usually exhibit morphological features similar to human breast cancer; as such, they have been proposed as a model for human breast cancers [15,16].

Using the WHO classification, the majority of the cases in our series were adenocarcinomas with predominant tubulopapillary and solid features. Of interest, cystic change was common and was seen in 33% of adenocarcinomas. When applying the Nottingham grading system that is widely accepted in human breast cancer, FMA grading was strongly influenced by generally high mitotic rates which averaged 37 mitoses, with a range of 7 to 90 per 10 high power fields (0.44 mm^2^ field diameter). In fact 29% of the FMAs had greater than 50 mitotic figures per 10 high power fields. This finding reflects the generally rapid growth that these tumors display. The distribution of 8% well differentiated, 50% moderately differentiated, and 42% poorly differentiated adenocarcinomas correlates well with a previous series of 55 FMAs [39] in which 14% were well differentiated, 60% were moderately differentiated and 27% were poorly differentiated. In that study, a good predictive value in relation to prognosis for well and poorly differentiated tumors was reported. The concordance of our series with the previous series also suggests that the Nottingham grading system applied to FMAs is reproducible across collections of cases.

Tumor necrosis is a common finding in rapidly growing neoplasms, whose growth often outpaces the ability of angiogenetic pathways to provide adequate vascular support. Indeed, in our study, with a high mitotic index in the majority of cases, necrosis was present in all but 3 cases, with those 3 tumors showing 20, 24 and 70 mitoses per 10 high power fields respectively. The presence of necrosis could not be associated with any other specific morphologic features.

Microcalcifications are often used as a surrogate marker for human breast cancers [40], allowing detection of such tumors mammographically by the detection of radiographic densities associated with these dystrophic calcifications. In human breast cancer, the majority of these represent calcium hydroxyapatite precipitates, which are readily identified by light microscopy with H&E staining. A second form of microcalcification composed of calcium oxalate accounts for up to 10% of cases with microcalcifications and is readily identified by polarization [41]. In our series, only two of the cases had calcium hydroxyapatite microcalcifications within the tumor, with no cases having calcium oxalate by polarized light microscopy. The relative lack of microcalfications in FMAs compared to human carcinomas may represent a species variation.

Lymphocytic infiltration was a common finding with 62.5% of the FMAs showing peritumoral infiltrates. Similarly, lymphocytic infiltration is not uncommon in human TNBCs, particularly in the basal-like subtype that was also overrepresented in this case series.

Estrogen and progesterone receptors are commonly expressed in human breast cancers, with ER expression being noted in 70-80% of cases [42]. However, about 15-20% of breast cancers in humans are negative for both ER and PR, typically in more poorly differentiated tumors [42]. Previous studies of FMAs have shown that ERs are expressed at much lower rates in FMAs and expression of PRs tends to slightly lower than in humans, but significantly higher than ER expression in most studies. In one study of 42 feline mammary carcinomas, 4 tumors (9.5%) were positive for ER by IHC while 28 tumors (66.7%) were positive for PR [21]. In a second similar study 6 of 27 tumors (22%) were positive for ER and this number correlated well with ER levels evaluated by a dextran-charcoal method [22]. Another study specifically compared ER and PR expression and found 5 of 25 cases (20%) of FMAs positive for ER by IHC and dextran-charcoal methods, and 9 of 25 (36%) of these cases were positive for PR [23]. Assay by dextran-charcoal method alone yielded similar findings, with 2 of 20 cases (10%) positive for ERs [19]. In our current series only 2 of 24 (8%) of cases were positive for ER, while 7 of 24 (29%) were positive for PR. Our data confirm the observation of lower levels of ER compared to PR in FMAs as well as lower ER and PR expression in FMAs compared to human breast cancer [20,21,23,42].

HER2 overexpression is seen in about 15-25% of human breast cancers, and is the basis for therapy with trastuzumab, an antibody based blocker of the HER2 membrane protein [43]. HER2 overexpression is commonly observed in cats with spontaneous feline mammary tumors [18,24,25,44,45], however, the reported percentages of HER2 overexpression in feline mammary tumors are highly variable between studies (5.5%-90%). In our study, 4 of 24 FMAs (17%) were immunopositive for HER2. Similar percentages (5.5%, 16% and 36%) of HER2 expression have been reported in some studies [18,24,25], while two other studies reported significantly higher (59%, 76.7%) percentages [44,45]. Variation of reported percentages is most likely based on selection of cases (benign and malignant as well as morphologic subtypes), technical factors and subjective interpretation. Variation in genetic backgrounds between different cat populations, such as incidence of breeds at different continents, may also contribute to such differences in reported percentages. Similar controversies regarding HER2 expression have been reported for human breast cancer, and the FDA approved HercepTest™ is recommended for an accurate and reliable evaluation of HER2 expression [25,46]. In this study, the standardized HercepTest™ was used for HER2 expression analysis and results were similar (17% versus 16%) to another study that applied the HercepTest™ scoring system [25].

Immunohistochemistry for ER, PR, and HER2 overexpression might also be negatively influenced by to short or prolonged fixation time or delayed fixation [47,48]. Since our cases represented surgical biopsy material, such negative influences are unlikely and the studied cases had been fixed between 24–48 hours according to recommended fixation times [49,50]. Furthermore, all of the cats in this study were family pets and most had been neutered/spayed at an early age.

In our study, 14 of the 24 (58%) cases were negative for ER and PR and for HER2 overexpression. In humans, such TNBC exhibit aggressive behavior and are further complicated by the lack of targeted therapy. FMAs appear to represent a rich source of TNBCs, and could provide a valuable model for the evaluation of effective therapeutic strategies for these tumors in humans. In human breast cancers, basal-like subtype tumors account for about 50-80% of all TNBC [51], which is similar to our series of FMAs with 79% of triple negative tumors exhibiting a basal-like subtype. Of particular interest, TNBCs include a subset of neoplasms with a basal-like subtype that are often associated with *BRCA1* mutations and are characterized by expression of genes usually found in basal or myoepithelial cells of the normal human breast [52]. Such cases can be identified by a series of specific surrogate basal markers, including CK5/6, CK17, CK14 and EGFR [51]. IHC expression of CK5/6 and/or EGFR in tumors negative for both ER and HER2/neu is a pragmatic and widely accepted method for diagnosing basal-like subtype TNBC [51].

This study represents an initial attempt to define the genetic alteration of triple negative FMAs with a basal-like subtype, in order to determine how this profile may differ from the genetic profiles of tumor of similar phenotype occurring in humans. We found no mutations or allelic losses in either the *BRCA1* or *BRCA2* gene. However only DNA from 5 cats could be amplified from archival formalin-fixed paraffin embedded material for genetic analysis. Larger numbers of samples need to be studied for a more comprehensive understanding of the potential role of BRCA mutations in FMAs of basal-like phenotype. It is also possible that epigenetic modifications which lead to *BRCA* gene silencing may play a role in these cases. In addition, since mutations in *BRCA1* and *BRCA2* were investigated within the locations reported in human breast cancer, cats may carry *BRCA1* and *BRCA2* mutations in different gene locations.

## Conclusion

FMAs have an aggressive biologic behavior, are of high histologic grade, and a high percentage of these tumors is triple negative, with about 80% displaying a basal-like subtype. Previous studies noted that estrogen and progesterone receptor negative proliferations occur spontaneously as purely intraepithelial lesions in feline mammary glands, and have suggested that these might be an effective model for evaluating the earliest stages of hormone receptor negative disease in humans [17]. In our study, we demonstrated a high incidence of triple negative basal-like FMAs that have a similar aggressive clinical behavior as their human counterparts and would be a valuable natural occurring animal model to study human TNBCs of basal-like subtype.

## Abbreviations

BRCA: Breast susceptibility cancer; LOH: Loss of heterozygosity; EGFR: Epidermal growth factor receptor; CK: Cytokeratins; ER: Estrogen receptor; PR: Progesterone receptor; IHC: Immunohistochemistry.

## Competing interests

The authors declare that they have no competing interests.

## Authors’ contributions

DAW conducted the histologic and immunohistochemical evaluation, TT carried out the DNA isolation, PCR, sequence analysis, and LOH analysis. VYG provided laboratory resources, directed the molecular study and supervised the molecular result analysis. MK selected the original case material, designed the study and together with DAW analyzed the histology and IHC results. DAW, TT and MK drafted the manuscript. All authors have read and approved the final manuscript.

## Pre-publication history

The pre-publication history for this paper can be accessed here:

http://www.biomedcentral.com/1471-2407/13/403/prepub

## References

[B1] Siegel R Ward E Brawley O Jemal A Cancer statistics, 2011: the impact of eliminating socioeconomic and racial disparities on premature cancer deaths CA Cancer J Clin 2011 61 212 236 10.3322/caac.20121 21685461

[B2] Morris SR Carey LA Molecular profiling in breast cancer Rev Endocr Metab Disord 2007 8 185 198 10.1007/s11154-007-9035-3 17464566

[B3] Sørlie T Perou CM Tibshirani R Aas T Geisler S Johnsen H Hastie T Eisen MB van de Rijn M Jeffrey SS Thorsen T Quist H Matese JC Brown PO Botstein D Lønning PE Børresen-Dale AL Gene expression patterns of breast carcinomas distinguish tumor subclasses with clinical implications Proc Natl Acad Sci USA 2001 98 10869 10874 10.1073/pnas.191367098 11553815 PMC58566

[B4] Nielsen TO Hsu FD Jensen K Cheang M Karaca G Hu Z Hernandez-Boussard T Livasy C Cowan D Dressler L Akslen LA Ragaz J Gown AM Gilks CB van de Rijn M Perou CM Immunohistochemical and clinical characterization of the basal-like subtype of invasive breast carcinoma Clin Cancer Res 2004 10 5367 5374 10.1158/1078-0432.CCR-04-0220 15328174

[B5] Cheang MC Voduc D Bajdik C Leung S McKinney S Chia SK Perou CM Nielsen TO Basal-like breast cancer defined by five biomarkers has superior prognostic value than triple-negative phenotype Clin Cancer Res 2008 14 1368 1376 10.1158/1078-0432.CCR-07-1658 18316557

[B6] Badve S Dabbs DJ Schnitt SJ Baehner FL Decker T Eusebi V Fox SB Ichihara S Jacquemier J Lakhani SR Palacios J Rakha EA Richardson AL Schmitt FC Tan PH Tse GM Weigelt B Ellis IO Reis-Filho JS Basal-like and triple-negative breast cancers: a critical review with an emphasis on the implications for pathologists and oncologists Mod Pathol 2011 24 157 167 10.1038/modpathol.2010.200 21076464

[B7] Haupt B Ro JY Schwartz MR Basal-like breast carcinoma: a phenotypically distinct entity Arch Pathol Lab Med 2010 134 130 133 20073617 10.5858/134.1.130

[B8] Anders CK Carey LA Biology, metastatic patterns and treatment of patients withtriple negative breast cancers Clin Breast Cancer 2009 9 Suppl 2 S73 S81 19596646 10.3816/CBC.2009.s.008 PMC2919761

[B9] Oakman C Viale G Di Leo A Management of triple negative breast cancer Breast 2010 19 312 321 10.1016/j.breast.2010.03.026 20382530

[B10] Dreyer G Vandorpe T Smeets A Forceville K Brouwers B Neven P Janssens H Deraedt K Moerman P Van Calster B Christiaens MR Paridaens R Wildiers H Triple negative breast cancer: clinical characteristics in the different histological subtypes Breast 2013 Feb 15 [Epub ahead of print] 10.1016/j.breast.2013.01.009 23416046

[B11] Ismail-Khan R Bui MM A review of triple-negative breast cancer Cancer Control 2010 17 173 176 20664514 10.1177/107327481001700305

[B12] Hayes H Milne K Mandell C Epidemiological features of feline mammary carcinoma Vet Rec 1981 30 476 479 7257136 10.1136/vr.108.22.476

[B13] Misdorp W Else RW Hellme’n E Lipscomb TP Histological classification of mammary tumors of the Dog and Cat Armed Forces Institute of Pathology in cooperation with the American Registry of Pathology and the World Health Organization Collaborating Center for Worldwide Reference on Comparative Oncology, vol. VII. 2nd series. Washington, DC 1999 1 59

[B14] Dias Pereira P Carvalheira J Gärtner F Cell proliferation in feline normal hyperplastic and neoplastic mammary tissue--an immunohistochemical study Vet J 2004 168 180 185 10.1016/j.tvjl.2003.10.018 15301767

[B15] Hahn K Bravo L Avenell J Feline breast carcinoma as a pathologic andtherapeutic model for human breast cancer In Vivo 1994 8 825 828 7727731

[B16] Zappulli V De Zan G Cardazzo B Bargelloni L Castagnaro M Feline mammary tumours in comparative oncology J Dairy Res 2005 72 Spec No 98 106 16180727 10.1017/s0022029905001263

[B17] Burrai GP Mohammed SI Miller MA Marras V Pirino S Addis MF Uzzau S Antuofermo E Spontaneous feline mammary intraepithelial lesions as a model for human estrogen receptor- and progesterone receptor-negative breast lesions BMC Cancer 2010 10 156 10.1186/1471-2407-10-156 20412586 PMC2873946

[B18] Rasotto R Caliari D Castagnaro M Zanetti R Zappulli V An Immunohistochemical study of HER2 expression in feline mammary tumours J Comp Pathol 2011 144 170 179 10.1016/j.jcpa.2010.08.010 20880546

[B19] Hamilton JM Else RW Forshaw P Oestrogen receptors in feline mammary carcinoma Vet Rec 1976 99 477 479 10.1136/vr.99.24.477 1014299

[B20] Rutteman GR Blankenstein MA Minke J Misdorp W Steroid receptors in mammary tumours of the cat Acta Endocrinol (Copenh) 1991 125 Suppl 1 32 37 1801501

[B21] Millanta F Calandrella M Bari G Niccolini M Vannozzi I Poli A Comparison of steroid receptor expression in normal, dysplastic, and neoplastic canine and feline mammary tissues Res Vet Sci 2005 79 225 232 10.1016/j.rvsc.2005.02.002 16054892

[B22] Mulas JM D l van Niel M Millán Y Blankenstein MA van Mil F Misdorp W Immunohistochemical analysis of estrogen receptors in feline mammary gland benign and malignant lesions: comparison with biochemical assay Domest Anim Endocrinol 2000 18 111 125 10.1016/S0739-7240(99)00067-3 10701768

[B23] Mulas JM D l Van Niel M Millán Y Ordás J Blankenstein MA Van Mil F Misdorp W Progesterone receptors in normal, dysplastic and tumourous feline mammary glands. Comparison with oestrogen receptors status Res Vet Sci 2002 72 153 161 10.1053/rvsc.2001.0542 12027597

[B24] De Maria R Olivero M Iussich S Nakaichi M Murata T Biolatti B Di Renzo MF Spontaneous feline mammary carcinoma is a model of HER2 overexpressing poor prognosis human breast cancer Cancer Res 2005 65 907 912 15705889

[B25] Ordas J Millan Y Dios R Reymundo C De las Mulas JM Proto-oncogene HER2 in normal, dysplastic and tumorous feline mammary glands: an immunohistochemical and chromogenic in situ hybridization study BMC Cancer 2007 7 179 184 10.1186/1471-2407-7-179 17880730 PMC2045669

[B26] Hughes K Dobson JM Prognostic histopathological and molecular markers in feline mammary neoplasia Vet J 2012 194 19 26 10.1016/j.tvjl.2012.05.008 22841451

[B27] Miki Y Swensen J Shattuck-Eidens D Futreal PA Harshman K Tavtigian S Liu Q Cochran C Bennett LM Ding W A strong candidate for the breast and ovarian cancer susceptibility gene BRCA1 Sciencen 1994 266 66 71 10.1126/science.7545954 7545954

[B28] Wooster R Bignell G Lancaster J Swift S Seal S Mangion J Collins N Gregory S Gumbs C Micklem G Identification of the breast cancer susceptibility gene BRCA2 Nature 1995 378 789 792 10.1038/378789a0 8524414

[B29] Miller BJ Wang D Krahe R Wright FA Pooled analysis of loss of heterozygosity in breast cancer: a genome scan provides comparative evidence for multiple tumor suppressors and identifies novel candidate regions Am J Hum Genet 2003 73 748 767 10.1086/378522 13680524 PMC1180599

[B30] Osorio A de la Hoya M Rodríguez-López R Martínez-Ramírez A Cazorla A Granizo JJ Esteller M Rivas C Caldés T Benítez J Loss of heterozygosity analysis at the BRCA loci in tumor samples from patients with familial breast cancer Int J Cancer 2002 99 305 309 10.1002/ijc.10337 11979449

[B31] Foulkes WD Stefansson IM Chappuis PO Bégin LR Goffin JR Wong N Trudel M Akslen LA Germline BRCA1 mutations and a basal epithelial phenotype in breast cancer J Natl Cancer Inst 2003 95 1482 1485 10.1093/jnci/djg050 14519755

[B32] Lakhani SR Van De Vijver MJ Jacquemier J Anderson TJ Osin PP McGuffog L Easton DF The pathology of familial breast cancer: predictive value of immunohistochemical markers estrogen receptor, progesterone receptor, HER2, and p53 in patients with mutations in BRCA1 and BRCA2 J Clin Oncol 2002 20 2310 2318 10.1200/JCO.2002.09.023 11981002

[B33] Veer LJ V ’t Dai H Van de Vijver MJ He YD Hart AA Mao M Peterse HL Van der Kooy K Marton MJ Witteveen AT Schreiber GJ Kerkhoven RM Roberts C Linsley PS Bernards R Friend SH Gene expression profiling predicts clinical outcome of breast cancer Nature 2002 415 530 536 10.1038/415530a 11823860

[B34] Elston CW Ellis IO Pathological prognostic factors in breast cancer. The value of histologic grade in breast cancer: experience from a large study with long term follow-up Histopathology 1991 19 403 410 10.1111/j.1365-2559.1991.tb00229.x 1757079

[B35] Zemke D Yamini B Yuzbasiyan-Gurkan V Mutations in the juxtamembrane domain of c-KIT are associated with higher grade mast cell tumors in dogs Vet Pathol 2002 39 529 535 10.1354/vp.39-5-529 12243462

[B36] Musolino A Bella MA Bortesi B Michiara M Naldi N Zanelli P Capelletti M Pezzuolo D Camisa R Savi M Neri TM Ardizzoni A BRCA mutations, molecular markers, and clinical variables in early-onset breast cancer: a population-based study Breast 2007 16 280 292 10.1016/j.breast.2006.12.003 17257844

[B37] Oetting WS Lee HK Flanders DJ Wiesner GL Sellers TA King RA Linkage analysis with multiplexed short tandem repeat polymorphisms using infrared fluorescence and M13 tailed primers Genomics 1995 30 450 458 10.1006/geno.1995.1264 8825630

[B38] Powell CA Klares S O’Connor G Brody JS Loss of heterozygosity in epithelial cells obtained by bronchial brushings: clinical utility in lung cancer Clin Cancer Res 1999 5 2025 2034 10473082

[B39] Castagnaro M Casalone C Bozzetta E De Maria R Biolatti B Caramelli M Tumour grading and the one-year post-surgical prognosis in feline mammary carcinomas J Comp Pathol 1998 119 263 275 10.1016/S0021-9975(98)80049-2 9807728

[B40] Morgan MP Cooke MM McCarthy GM Microcalcifications associated with breast cancer: an epiphenoenon or biologically significant feature of selected tumors? J Mammary Gland Biol Neoplasia 2005 10 181 187 10.1007/s10911-005-5400-6 16025224

[B41] Tornos C Silva E el Naggar A Pritzker KP Calcium oxalate crystals in breast biopsies. The missing microcalcifications Am J Surg Pathol 1990 14 961 968 10.1097/00000478-199010000-00010 1698342

[B42] Zafrani B Aubriot MH Mouret E De Crémoux P De Rycke Y Nicolas A Boudou E Vincent-Salomon A Magdelénat H Sastre-Garau X High sensitivity and specificity of immunohistochemistry for the detection of hormone receptors in breast carcinoma: comparison with biochemical determination in a prospective study of 793 cases Histopathology 2000 37 536 545 10.1046/j.1365-2559.2000.01006.x 11122436

[B43] Lal P Tan LK Chen B Correlation of HER2 status with estrogen and Progesterone receptors and histologic features in 3,655 invasive breast carcinomas Am J Clin Pathol 2005 123 541 546 10.1309/YMJ3A83TB39MRUT9 15743737

[B44] Winston J Craft DM Scase TJ Bergman PJ Immunohistochemical detection of HER2/neu expression in spontaneous feline mammary tumours Vet Comp Oncol 2005 3 8 15 10.1111/j.1476-5810.2005.00063.x 19379209

[B45] Millanta F Calandrella M Citi S Della Santa D Poli A Overexpression of HER2 in feline invasive mammary carcinomas: an immunohistochemical survey and evaluation of its prognostic potential Vet Pathol 2005 42 30 34 10.1354/vp.42-1-30 15657269

[B46] Birner P Oberhuber G Stani J Reithofer C Samonigg H Hausmaninger H Kubista E Kwasny W Kandioler-Eckersberger D Gnant M Jakesz R Austrian Breast & Colorectal Cancer Study Group Evaluation of the United States Food and Drug Administration-approved scoring and test system of HER2 protein expression in breast cancer Clin Cancer Res 2001 7 1669 1675 11410505

[B47] Khoury T Sait S Hwang H Chandrasekhar R Wilding G Tan D Kulkarni S Delay to formalin fixation effect on breast biomarkers Mod Pathol 2009 22 1457 1467 10.1038/modpathol.2009.117 19734848

[B48] Hammond MEH Hayes DF Dowsett M Allred DC Hagerty KL Badve S Fitzgibbons PL Francis G Goldstein NS Hayes M Hicks DG Lester S Love R Mangu PB McShane L Miller K Osborne CK Paik S Perlmutter J Rhodes A Sasano H Schwartz JN Sweep FC Taube S Torlakovic EE Valenstein P Viale G Visscher D Wheeler T Williams RB American Society of Clinical Oncology/College of American Pathologists guideline recommendations for immunohistochemical testing of estrogen and progesterone receptors in breast cancer J ClinOncol 2010 28 2784 2795 10.1200/JCO.2009.25.6529 PMC2881855 20404251

[B49] Ibarra JA Rogers LW Kyshtoobayeva A Bloom K Fixation time does not affect the expression of estrogen receptor Am J Clin Pathol 2010 133 747 755 10.1309/AJCPPIUHS4GVAR0I 20395521

[B50] Arber DA Effect of prolonged formalin fixation on the immunohistochemical reactivity of breast markers Appl Immunhohistochem Mol Morphol 2002 10 183 186 10.1097/00022744-200206000-00015 12051639

[B51] Rakha EA Elsheikh SE Aleskandarany MA Habashi HO Green AR Powe DG El-Sayed ME Benhasouna A Brunet JS Akslen LA Evans AJ Blamey R Reis-Filho JS Foulkes WD Ellis IO Triple-negative breast cancer: distinguishing between basal and nonbasal subtypes Clin Cancer Res 2009 15 2302 2310 10.1158/1078-0432.CCR-08-2132 19318481

[B52] Diaz LK Cryns VL Symmans WF Sneige N Triple negative breast carcinoma and the basal phenotype: from expression profiling to clinical practice Adv Anat Pathol 2007 14 419 430 10.1097/PAP.0b013e3181594733 18049131

